# Interacting Multiple Model Estimators for Fault Detection in a Magnetorheological Damper

**DOI:** 10.3390/s24010251

**Published:** 2023-12-31

**Authors:** Andrew Sanghyun Lee, Yuandi Wu, Stephen Andrew Gadsden, Mohammad AlShabi

**Affiliations:** 1 College of Engineering and Physical Sciences, University of Guelph, Guelph, ON N1G 2W1, Canada; alee32@uoguelph.ca; 2 Department of Mechanical Engineering, McMaster University, Hamilton, ON L8S 4L8, Canada; wuy187@mcmaster.ca; 3 Department of Mechanical and Nuclear Engineering, University of Sharjah, Sharjah 27272, United Arab Emirates; malshabi@sharjah.ac.ae

**Keywords:** fault detection, estimation theory, interacting multiple model (IMM), extended Kalman filter (EKF), extended sliding innovation filter (ESIF), unscented Kalman filter (UKF)

## Abstract

This paper proposes a novel estimator for the purpose of fault detection and diagnosis. The interacting multiple model (IMM) strategy is effective for estimating the behaviour of systems with multiple operating modes. Each mode corresponds to a distinct mathematical model and is subject to a filtering process. This paper applies various model-based filters in combination with the IMM strategy. One such estimator employs the recently introduced extended sliding innovation filter (ESIF) known as the IMM-ESIF. The ESIF is an extension of the sliding innovation filter for nonlinear systems based on the sliding mode concept. In the presence of modeling uncertainties, the ESIF has been proven to be more robust compared to methods such as the extended Kalman filter (EKF). The novel IMM-ESIF strategy is also compared with the IMM strategy, which incorporates the unscented Kalman filter (UKF), referred to herein as IMM-UKF. While EKF uses Taylor series approximation to linearize the system model, the UKF uses sigma point to calculate the system’s mean and covariance. The methods were applied to an experimental magnetorheological (MR) damper setup, which was designed for testing control and estimation theory. Magnetorheological dampers exhibit a diverse array of applications in the automotive and aerospace sectors, with particular relevance to attenuating vibrations through adaptive suspension systems. Applied to a magnetorheological (MR) damper with distinct operating modes determined by the damper’s current, the results showcase the effectiveness of IMM-ESIF. In mixed operational conditions, IMM-ESIF demonstrates a notable 80% to 90% reduction in estimation error compared to its counterparts. Furthermore, it exhibits a 4% to 5% enhancement in correctly classifying operational modes, establishing IMM-ESIF as a promising and efficient alternative for adaptive estimation in electromechanical systems. The improved accuracy in estimating the system’s behaviour, even amidst uncertainties and mixed operational scenarios, signifies the potential of IMM-ESIF to significantly enhance the overall robustness and efficiency of estimations.

## 1. Introduction

Electromechanical systems commonly exhibit distinct operational modes due to various influences such as design specifications, environmental conditions, or the occurrence of faults. When these different operational modes are amenable to modeling, the application of adaptive estimation techniques can enhance the accuracy of estimation and facilitate fault detection. In the context of magnetorheological dampers, fluctuations in temperature and power supply failures can induce substantial alterations in the system’s behaviour. These abrupt and unforeseeable changes introduce a notable degree of uncertainty. In the process of developing filtering strategies for forecasting the system’s output force, adaptive algorithms can integrate multiple models to mitigate estimation errors.

Multiple model (MM) algorithms operate on a Bayesian framework to facilitate adaptive estimation. Various forms of the algorithm include static MM, dynamic MM, generalized pseudo-Bayesian MM, and interacting MM (IMM) [1,2,3,4,5,6,7,8]. The Bayesian premise of the MM methods involves updating the probability of a system existing in a specific mode following the acquisition of a new measurement. The algorithms incorporate a finite number of modes and use state estimates to calculate the probability associated with each mode.

The interacting multiple model Kalman filter (IMM-KF) is a widely adopted multiple model (MM) technique. This approach incorporates a set of Kalman filters (KFs), with the quantity of KFs aligning with the number of concurrent operating system models. The KF is preferred for its capacity to deliver optimal state estimates and its straightforward process for calculating corrective gains. Nevertheless, it is important to note that this method generates accurate state estimates or precise models only for linear systems that exhibit white noise (i.e., noise with a zero mean and a normal distribution) [9]. The Kalman gain is calculated by minimizing the trace of the *a priori* (predicted) state error covariance, which is a measure of the estimation error distribution [9,10,11]. The KF has been used in several applications, such as signal processing, fault detection, and target tracking [9]. However, the stability of the estimates may be compromised in the presence of disturbances, nonlinearities, and modeling uncertainties.

In nature, most systems exhibit some form of nonlinear behaviour. The extended Kalman filter (EKF) approximates the nonlinear process through local linearization around the a priori state estimate [9]. A first-order Taylor series of the nonlinear system model and measurement process is employed to compute the corresponding Jacobian matrices. The Jacobians may then be applied to the states and their covariance to calculate the corrective Kalman gain. However, the EKF estimates may diverge from the true state trajectory if the system is highly nonlinear [12].

Another method of capturing nonlinear behaviour is sampling. The unscented Kalman filter (UKF) generates samples from a probability distribution of states propagated through the system model known as sigma points [13]. The unscented transform is a deterministic sampling method that selects a minimal number of sample points around a mean, which, in this context, refers to the previous state estimate [9]. The mean and covariance of the projected points can be approximated using Monte Carlo sampling. Unlike the EKF, the UKF can approximate the updated statistical state mean and state error covariance up to the third order for nonlinear processes [12]. In addition, the UKF does not require taking partial derivatives of the system model or measurement process. However, the unscented transform generally comes at a higher computational cost when compared to the EKF [9].

Variable structure control and a sliding mode controller framework were used in the formulation of sliding mode observers (SMOs) [13]. The innovation is used to determine the observer gain that ideally forces the error surface towards the origin [13]. SMOs define a sliding surface, or hyperplane, in order to apply a discontinuous switching force [14]. This practice maintains the estimated values within the confines of the sliding surface. In 2007, the smooth variable structure filter (SVSF) was presented based on SMO concepts [15].

The measurement error and a switching term are used to calculate the SVSF gain [15,16]. The state estimates are bounded to the trajectory of the true state values by the switching term, thereby enhancing the stability of the estimation process. While classical model-based filters incorporate the state error covariance in the corrective gain calculation, the original formulation of the SVSF did not. The corrective gain was later expanded by minimizing the state error covariance. This optimization process results in a time-varying smoothing boundary layer [15]. The boundary layer widths vary depending on the degree of uncertainty inherent in the estimation process. In addition, the SVSF has been improved through the incorporation of a chattering function for higher-order solutions and fault detection [13,16,17,18].

The sliding innovation filter (SIF) was first presented in 2020 based on SMOs as an improvement over the SVSF [19]. The SIF retains robustness to uncertainties but uses a more concise gain structure and achieves higher estimation accuracy. This paper introduces a novel IMM strategy, which leverages an extension of the SIF tailored for the treatment of nonlinear systems. This extended adaptation is termed the extended sliding innovation filter (ESIF). Similar to the EKF, the ESIF utilizes the Jacobian matrix for linear approximation of the system to calculate the a priori state error covariance. The IMM algorithm is combined with the ESIF to form the IMM-ESIF, as demonstrated in [20].

The following work will detail an approach to fault detection and diagnosis through the development of a novel IMM-ESIF estimator. The proposed estimation strategies are applied on a magnetorheological (MR) damper, which was built specifically for creating a benchmark platform to test out new control and estimation theory. The MR damper setup, which will be described later in the paper, can be modelled and operated according to a finite number of distinct mathematical models. Leveraging the proven effectiveness of the IMM strategy for systems with multiple operating modes, the proposed estimator integrates the recently introduced ESIF due to its heightened robustness in addressing modeling uncertainties. The IMM strategy enhances fault detection in MR dampers by explicitly modeling both fault and nominal modes within the system. In the context of MR dampers, the IMM strategy considers multiple dynamic models that capture the variations in behaviour associated with fault conditions and normal operation. Each dynamic model corresponds to a specific mode, representing either a fault scenario or the nominal state of the damper. The IMM strategy incorporates a filtering process that utilizes probability outputs to estimate the likelihood of being in a particular mode at any given time. By considering the probabilities associated with each dynamic model, the IMM approach offers a nuanced understanding of the system’s behaviour, enabling more accurate fault detection. This flexibility and adaptability make the IMM strategy effective for systems such as magnetorheological dampers, where multiple operating modes can significantly influence performance, and a single fixed model might be insufficient to capture the dynamic behaviour accurately. The probabilistic nature of IMM allows for a robust estimation process that accounts for uncertainties and mode transitions, enhancing its capability for fault detection in complex and variable systems.

A meticulous comparison of the IMM-ESIF with IMM-EKF and IMM-UKF is performed on experimental data from a physical MR Damper test bench, showcasing its notably superior performance in estimation accuracy and mode classification, particularly in the challenging scenario of mixed operational conditions. It was found that the IMM-ESIF exhibits a significant reduction in estimation error and demonstrates improvements in its capability to correctly classify operational modes compared to its counterparts. From the results, the novel IMM-ESIF emerges as a promising and efficient alternative for fault detection and diagnosis in electromechanical systems, setting a new standard for adaptive estimation strategies.

The present study is structured as follows. The estimation methods employed herein are expounded in Section 2, followed by the IMM algorithm in Section 3. A comprehensive exposition of the experimental configuration is provided in Section 4. The formulation of the mathematical model governing the MR damper is elaborated in Section 5, while empirical findings are presented in Section 6. Finally, the conclusions of this paper are drawn in Section 7.

## 2. Estimation Methods

### 2.1. Extended Kalman Filter

While the KF produces the optimal estimate for linear systems with white noise, the majority of systems in nature exhibit nonlinear behaviour. The states and measurements are determined by the nonlinear functions as follows:

(1) $$x_{k+1}=f\left(x_{k},u_{k}\right)+w_{k},$$

(2) $$z_{k+1}=h\left(x_{k+1}\right)+v_{k+1},$$

where *f* and *h* are the nonlinear system process and measurement functions, respectively, $u_{k}$ is the input, and $w_{k}$ and $v_{k+1}$ are the system and measurement noise, respectively.

The EKF exhibits a similar structure to the conventional Kalman Filter (KF), with the exception of variances in the system and measurement matrices. The nonlinear systems and measurement functions cannot be applied to the covariances directly. Instead, linear approximations of the nonlinear functions *f* and *h* are generated using a first-order Taylor series. The resulting Jacobian matrix can then be applied to the state error covariance matrix. In the case of highly nonlinear systems, it is observed that the utilization of a first-order Taylor series may result in an inaccurate approximation of the system’s behaviour. This inaccuracy has the potential to lead to instability in the estimation process [21,22]. The first-order partial derivatives of the nonlinear functions with respect to the states produce the Jacobian of the system function $F_{k}$ and the Jacobian of the measurement process $H_{k+1}$ as follows:

(3) $$F_{k}={\left.\frac{\partial f(x)}{\partial x}\right|}_{x={\hat{x}}_{k|k},u_{k}},$$

(4) $$H_{k+1}={\left.\frac{\partial h(x)}{\partial x}\right|}_{x={\hat{x}}_{k+1|k}}.$$

The system and measurement functions are linearized around the state estimate from the preceding time step [23]. As the linearization serves as an approximation of the system’s behaviour, the EKF no longer yields the optimal state estimates [23]. The prediction stage of the EKF consists of the a priori state estimate ${\hat{x}}_{k+1|k}$, which uses the nonlinear system model, as well as the state error covariance $P_{k+1|k}$, which uses the Jacobian of the system model. The prediction stage equations are given as follows:

(5) $${\hat{x}}_{k+1|k}=f\left({\hat{x}}_{k|k},u_{k}\right),$$

(6) $$P_{k+1|k}=F_{k}P_{k|k}F_{k}^{T}+Q_{k},$$

where ${\hat{x}}_{k|k}$ is the previous state estimate, $u_{k}$ is the system input, $P_{k|k}$ is the previous state error covariance, and $Q_{k}$ is the system noise covariance. The matrix transpose operator is denoted by *T*. The innovation ${\tilde{z}}_{k+1}$ is calculated based on the nonlinear measurement function h given by Equation (7). The innovation covariance matrix $S_{k+1}$, extended Kalman gain $K_{k+1}$, and a posteriori state error covariance $P_{k+1|k+1}$ all utilize the Jacobian of the measurement function $H_{k+1}$, as shown in Equations (8), (9) and (11). The innovation covariance $S_{k+1}$ is used to calculate the extended Kalman gain $K_{k+1}$. This is applied to the innovation ${\tilde{z}}_{k+1}$ to update the a priori state estimate ${\hat{x}}_{k+1|k}$ and produce the a posteriori state estimate ${\hat{x}}_{k+1|k+1}$, as shown in Equation (10). The entirety of the update stage is given by the following [23]:

(7) $${\tilde{z}}_{k+1}=z_{k+1}-h\left({\hat{x}}_{k+1|k}\right),$$

(8) $$S_{k+1}=H_{k+1}P_{k+1|k}H_{k+1}^{T}+R_{k+1},$$

(9) $$K_{k+1}=P_{k+1|k}H_{k+1}^{T}S_{k+1}^{-1},$$

(10) $${\hat{x}}_{k+1|k+1}={\hat{x}}_{k+1|k}+K_{k+1}{\tilde{z}}_{k+1},$$

(11) $$P_{k+1|k+1}=\left(w-K_{k+1}H_{k+1}\right))P_{k+1|k},$$

where $R_{k+1}$ is the measurement noise covariance, and *I* is the identity matrix. Similar to the KF, the EKF is known for its straightforward implementation [22]. However, special consideration should be given to nonlinear systems that cannot be approximated accurately by a first-order Taylor series.

### 2.2. Unscented Kalman Filter

An alternative approach for addressing nonlinearities involves employing statistical linear regression of sample points projected using the nonlinear system model [24]. The unscented Kalman filter (UKF) is a popular formulation of the sigma-point Kalman filter (SPKF). The UKF generates sigma points based on the previous state estimate and covariances. The sigma points are then projected using the nonlinear system model to form the a priori state estimate and state error covariance in a process known as the unscented transform [25,26]. Additionally, the points are also projected using the nonlinear measurement function as well. This method eliminates the necessity for linearization and generally yields a more precise estimation compared to the Jacobian approximation for the nonlinear system [21,25,27,28].

The UKF algorithm is detailed in the following equations [29]. Given a state space with dimension *n*, the state $x_{k}$ can be represented with 2n+1 sigma points denoted by *X*. The sigma points have a mean of ${\hat{x}}_{k|k}$ and a covariance of $P_{k|k}$. The initial sigma point $X_{0,k|k}$ and corresponding weight $W_{0}$ are given as follows:

(12) $$X_{0,k|k}={\hat{x}}_{k|k},$$

(13) $$W_{0}=\frac{\kappa }{n+\kappa },$$

where κ is a design parameter. The next 2n number of sigma points are calculated as follows:

(14) $$X_{i,k|k}={\hat{x}}_{k|k}+{\left(\sqrt{\left(n+\kappa \right)P_{k|k}}\right)}_{i},$$

(15) $$W_{i}=\frac{1}{2(n+\kappa )},$$

where the value $X_{i,k|k}$ is the *i*th sigma point and $W_{i}$ is the weight that is associated with the *i*th sigma point [30]. The sigma points are projected $\left({\hat{X}}_{i,k+1|k}\right)$ through the nonlinear system function *f* and added together with their corresponding weights to produce the a priori state estimate ${\hat{x}}_{k+1|k}$ as follows [9]:

(16) $${\hat{X}}_{i,k+1|k}=f\left(X_{i,k|k},u_{k}\right),$$

(17) $${\hat{x}}_{k+1|k}=\sum_{i=0}^{2n}W_{i}{\hat{X}}_{i,k+1|k}.$$

The previous calculations are used to calculate the a priori state error covariance as follows [9]:

(18) $$P_{k+1|k}=\sum_{i=0}^{2n}W_{i}\left({\hat{X}}_{i,k+1|k}-{\hat{x}}_{k+1|k}\right){\left({\hat{X}}_{i,k+1|k}-{\hat{x}}_{k+1|k}\right)}^{T}+Q_{k}.$$

The sigma points are also propagated through the nonlinear measurement function. Unlike the KF and EKF, the UKF calculates a predicted measurement ${\hat{z}}_{k+1|k}$, which is used to produce the innovation covariance $P_{zz.k+1|k}$.

(19) $${\hat{Z}}_{i,k+1|k}=h\left({\hat{X}}_{i,k+1|k},u_{k}\right),$$

(20) $${\hat{z}}_{k+1|k}=\sum_{i=0}^{2n}W_{i}{\hat{Z}}_{i,k+1|k},$$

(21) $$P_{zz,k+1|k}=\sum_{i=0}^{2n}W_{i}\left({\hat{Z}}_{i,k+1}-{\hat{z}}_{k+1|k}\right){\left({\hat{Z}}_{i,k+1}-{\hat{z}}_{k+1|k}\right)}^{T}+R_{k+1}.$$

The cross-covariance (with respect to the state and measurement) is calculated as follows [9]:

(22) $$P_{xz,k+1|k}=\sum_{i=0}^{2n}W_{i}\left({\hat{X}}_{i,k+1}-{\hat{x}}_{k+1|k}\right){\left({\hat{Z}}_{i,k+1}-{\hat{z}}_{k+1|k}\right)}^{T}.$$

The cross-covariance $P_{xz,k+1|k}$ and innovation covariance $P_{zz,k+1|k}$ are combined to produce the corrective gain $K_{k+1}$ as follows:

(23) $$K_{k+1}=P_{xz,k+1|k}P_{zz,k+1|k}^{-1}.$$

To conclude the updated state of the UKF, the a posteriori state estimate and a posteriori state error covariance are given as follows [9]:

(24) $${\hat{x}}_{k+1|k+1}={\hat{x}}_{k+1|k}+K_{k+1}\left(z_{k+1}-{\hat{z}}_{k+1|k}\right),$$

(25) $$P_{k+1|k+1}=P_{k+1|k}-K_{k+1}p_{zz,k+1|k}K_{k+1}^{T}.$$

In the case of the UKF, there is a trade-off between computational cost and estimation accuracy. While the EKF only propagates a single state estimate through a nonlinear process, the UKF uses 2n+1 sigma points to achieve a more accurate state estimate and state error covariance. The performance of the UKF is akin to that of the EKF for systems exhibiting mild nonlinearity, but it demonstrates superior performance when dealing with nonlinear processes that cannot be suitably approximated using a first-order Taylor series [11].

### 2.3. Extended Sliding Innovation Filter

**Figure 1:**
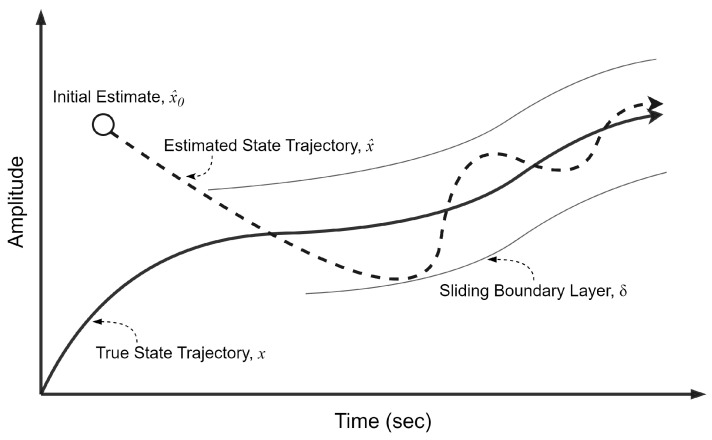
The SIF concept depicting the effects of the switching gain structure and sliding boundary layer, adapted from [19].

The SIF is a Bayesian, model-based estimator based on SMO concepts. The SIF corrective gain is calculated using the measurement matrix, innovation signifying the measurement error, as well as the sliding boundary layer. It is noted that this boundary layer remains constant in the conventional formulation of the SIF. The fixed boundary layer represents an upper limit of potential noise/disturbances and modeling uncertainty [19]. The initial estimate is forced towards the sliding boundary layer, or hyperplane. However, if the estimate is already within the hyperplane layer, the corrective gain forces the estimates to switch around the true state trajectory, as shown in Figure 1.

For a linear system, the prediction stage is identical to the EKF in Section 2.1 as follows:

(26) $${\hat{x}}_{k+1|k}=f\left({\hat{x}}_{k|k},u_{k}\right),$$

(27) $$P_{k+1|k}=F_{k}P_{k|k}F_{k}^{T}+Q_{k},$$

(28) $${\tilde{z}}_{k+1|k}=z_{k+1}-C{\hat{x}}_{k+1|k}.$$

However, the measurement process *h* was considered to be linear and constant for the purpose of this research. Thus, measurement matrix *C* was used instead of the Jacobian $H_{k+1}$. The extended sliding innovation filter (ESIF) is a formulation of the SIF for nonlinear system models and measurement processes. The ESIF corrective gain $K_{k+1}i$ is calculated using the measurement matrix *C*, innovation ${\tilde{z}}_{k+1|k}$ and fixed sliding boundary layer δ. The corrective gain is applied to the innovation stage to calculate the a posteriori state estimate in a similar fashion to the EKF. In addition, the a posteriori state error covariance follows the EKF formulation as well. The entirety of the update stage is given as follows [19]:

(29) $$K_{k+1}=C^{+}\bar{sat}\left(\frac{\left|{\tilde{z}}_{k+1|k}\right|}{\delta }\right),$$

(30) $${\hat{x}}_{k+1|k+1}={\hat{x}}_{k+1|k}+K_{k+1}{\tilde{z}}_{k+1|k},$$

(31) $$P_{k+1|k+1}=\left(I-K_{k+1}C^{+}\right)P_{k+1|k}{\left(I-K_{k+1}C^{+}\right)}^{T}+K_{k+1}R_{k+1}K_{k+1}^{T},$$

where $C^{+}$ is the pseudoinverse of the measurement matrix, $\bar{sat}$ is the diagonal matrix of the saturated vector values, and $\left|{\tilde{z}}_{k+1|k}\right|$ refers to the absolute innovation value [19]. The adjustment of the sliding boundary layer term is accomplished through manual tuning informed by an understanding of the system, encompassing factors such as noise and modeling uncertainty, or via alternative optimization techniques, with the objective of minimizing the estimation error [1]. The SIF estimation process can be summarized by Equation (26) through (31). Proof of stability for the SIF is provided in [19]. The updated innovation was used to define a Lyapunov function in order to prove that the estimation error is bounded.

## 3. Proposed IMM-SIF

The interacting multiple model (IMM) method incorporates a finite number of models and filtering strategies that run in parallel. Each filter associated with a specific model generates its distinct state estimate, state error covariance, and an indication of the model’s correctness. The likelihood is a function of the innovation (measurement error) and its covariance. This indication is contingent on the innovation (measurement error) and its covariance. Subsequently, these indications are leveraged to compute mode probabilities, which signify the likelihood of the system adopting a particular mode based on the current information.

The IMM method’s access to additional modeling information presents a clear advantage over single-model strategies [31]. Combining the IMM with the ESIF adds stability and robustness while increasing adaptability and accuracy with access to multiple models. In this paper, the efficacy of this strategy is evaluated against previous IMM strategies, such as the IMM-EKF and IMM-UKF, when applied to a highly nonlinear MR damper system.

**Figure 2:**
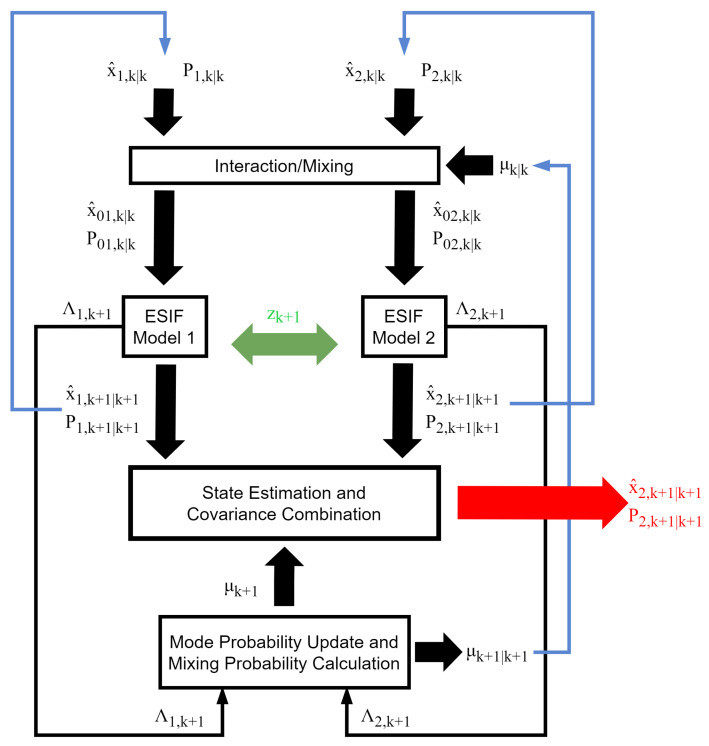
Overview of the proposed IMM-ESIF algorithm.

The IMM-ESIF algorithm is shown in Figure 2. The green arrows indicate measurement input, the blue arrows indicate recursion, and the red arrow indicates the overall IMM-ESIF output. A number of SIFs equivalent to the number of models are run in parallel. While Figure 2 shows two models for conciseness, there is no limit to the number of models that can be incorporated. However, it should be noted that processing time scales linearly with each additional model. The IMM-ESIF estimator consists of five steps: mixing probability calculation, ESIF mode-matched filtering, mode probability update, and a combination of the state estimate and covariance.

The mixing probabilities $\mu_{i|j,k|k}$ represent the probability of the system in mode *i* and switching to mode *j* at the next time step. The mixing probabilities are calculated as follows [8]:

(32) $$\mu_{i|j,k|k}=\frac{1}{{\bar{c}}_{j}}p_{ij}\mu_{i,k},$$

(33) $${\bar{c}}_{j}=\sum_{i=1}^{r}p_{ij}\mu_{i,k},$$

where $p_{ij}$ is the mode transition probability, which is a design parameter, $\mu_{ik}$ is the probability of the system existing in mode *i*, and *r* is the number of system modes. The previous mode matched state ${\hat{x}}_{i,k|k}$ and covariance $P_{i,k|k}$ are used to calculate the mixed initial conditions state ${\hat{x}}_{0j,k|k}$ and covariance $P_{0j,k|k}$ for the filter matched to mode *j* as follows [8]:

(34) $${\hat{x}}_{0j,k|k}=\sum_{i=1}^{r}{\hat{x}}_{ij,k|k}\mu_{i|j,k|k},$$

(35) $$P_{0j,k|k}=\sum_{i=0}^{r}\mu_{i|j.k|k}\left\{P_{i,k|k}+\left({\hat{x}}_{i,k|k}-{\hat{x}}_{0,k|k}\right){\left({\hat{x}}_{i,k|k}-{\hat{x}}_{0,k|k}\right)}^{T}\right\}.$$

These mixed initial conditions are then fed into the filters matched to mode *j*. Each ESIF uses the measurement $z_{k+1}$ as well as any system inputs $u_{k}$ to calculate the updated states and corresponding state error covariance. The initial state estimate ${\hat{x}}_{0j,k|k}$ and corresponding state error covariance $P_{0j,k|k}$ for each mode *j* are used to calculate the a priori states ${\hat{x}}_{j,k+1|k}$ error covariance $P_{j,k+1|k}$ as follows:

(36) $${\hat{x}}_{j,k+1|k}=f_{j}\left({\hat{x}}_{0j,k|k},u_{k}\right),$$

(37) $$P_{j,k+1|k}=F_{j}P_{0j,k|k}F_{j}^{T}+Q_{k},$$

where $f_{j}$ is the nonlinear system state equations of mode *j* and $F_{j}$ is the Jacobian matrix of said equations.

The mode-matched innovation covariance $S_{j,k+1|k}$ and mode-matched a priori measurement error $e_{j,z,k+1|k}$ are calculated as follows [8]:

(38) $$S_{j,k+1|k}=C_{j}P_{j,k+1|k}C_{j}^{T}+R_{k+1},$$

(39) $$e_{j,z,k+1|k}=z_{k+1}-C_{j}{\hat{x}}_{j,k+1|k},$$

where the measurement matrix $C_{j}$ is considered linear and constant for the purposes of this paper.

The update stage is described by the following four equations. The mode-matched ESIF gain $K_{j,k+1}$ is calculated via Equation (40) and used to update the state estimate ${\hat{x}}_{j,k+1|k+1}$ via Equation (41).

(40) $$K_{i,k+1}=H_{j}^{+}\bar{sat}\left(\frac{\left|e_{j,z,k+1|k}\right|}{\delta }\right),$$

(41) $${\hat{x}}_{j,k+1|k+1}={\hat{x}}_{j,k+1|k}+K_{j,k+1}e_{j,z,k+1|k}.$$

The updated state error covariance matrix $P_{j,k+1|k+1}$ is generated via Equation (42) and is used to produce the a posteriori measurement error $e_{j,z,k+1|k+1}$, as illustrated in Equation (43).

(42) $$P_{j,K+1|k+1}=\left(I-K_{j,k+1}C_{j}\right)P_{j,k+1|k}{\left(I-K_{j,k+1}C_{j}\right)}^{T}+K_{j,k+1}R_{k+1}K_{j,k+1}^{T},$$

(43) $$e_{j,z,k+1|k+1}=z_{k+1}-H_{j}{\hat{x}}_{j,k+1|k+1}.$$

Using the mode-mode matched innovation matrix $S_{j,k+1|k}$ and the mode-matched updated measurement error $e_{j,z,k+1|k}$, a corresponding likelihood function $\Lambda_{j,k+1}$ is calculated as follows [8]:

(44) $$\Lambda_{j,k+1}=N\left(z_{k+1};e_{j,z,k+1|k},S_{j,k+1|K}\right).$$

The likelihood is calculated by applying measurement $z_{k+1}$ to a Gaussian probability density function with mean $e_{j,z,k+1|k}$ and covariance $S_{j,k+1|k}$. The likelihood can be rewritten as the following Equation [8]:

(45) $$\Lambda_{j,k+1}=\frac{1}{\sqrt{\left|2\pi S_{j,k+1|k}\right|}}exp\left(\frac{-\frac{1}{2}e_{j,z,k+1|k}^{T}e_{j,z,k+1|k}}{S_{j,k+1|k}}\right).$$

The mode-matched likelihood function $\Lambda_{j,k+1}$ is then used to update the mode probability $\mu_{i,k}$, as shown [8]:

(46) $$\mu_{i,k}=\frac{1}{c}\Lambda_{j,k+1}\sum_{i=1}^{r}p_{ij}\mu_{i,k},$$

where the normalizing constant *c* is defined as follows [8]:

(47) $$c=\sum_{j=1}^{r}\Lambda_{j,k+1}\sum_{j=1}^{r}p_{ij}\mu_{i,k}.$$

Finally, the IMM-ESIF outputs the overall state estimates ${\hat{x}}_{k+1|k+1}$ and corresponding state error covariance $P_{k+1|k+1}$, which are calculated as follows [8]:

(48) $${\hat{x}}_{k+1|k+1}=\sum_{j=1}^{r}\mu_{i,k+1}{\hat{x}}_{j,k+1|k+1},$$

(49) $$P_{k+1|k+1}=\sum_{j+1}^{r}\mu_{i,k+1}\left\{P_{i,k+1|k+1}+\left({\hat{x}}_{j,k+1|k+1}-{\hat{x}}_{k+1|k+1}\right){\left({\hat{x}}_{j,k+1|k+1}-{\hat{x}}_{k+1|k+1}\right)}^{T}\right\}.$$

The formulation of the IMM-ESIF can be summarized by Equations (32)–(49). Note that the estimator’s overall output ${\hat{x}}_{k+1|k+1}$ from (48) and $P_{k+1|k+1}$ from (49) are not used in the algorithm recursions [8]. The IMM-EKF and IMM-UKF follow a similar process, with the primary difference being their respective corrective gain calculations.

## 4. Experimental Setup

The primary component of the experimental setup utilized in this study is the RD-8041-1 MR damper, procured from LORD corporation. MR dampers exhibit a diverse array of applications in the automotive and aerospace sectors, with particular relevance to attenuating vibrations through adaptive suspension systems [32]. A typical MR damper consists of the MR fluid itself, housing, piston, diaphragm, and magnetic coil [33]. The manipulation of the damper’s performance involves the supply of an electrical current to modulate the viscosity of the MR fluid, thereby elevating the damping force. This variation in viscosity arises from the repositioning of ferromagnetic particles dispersed within the fluid. In the presence of a magnetic field, these particles align to create linear chain structures [33]. As the MR damper is driven, the MR fluid moves between different chambers via small orifices in the piston assembly and converts mechanical energy into friction losses [33].

**Figure 3:**
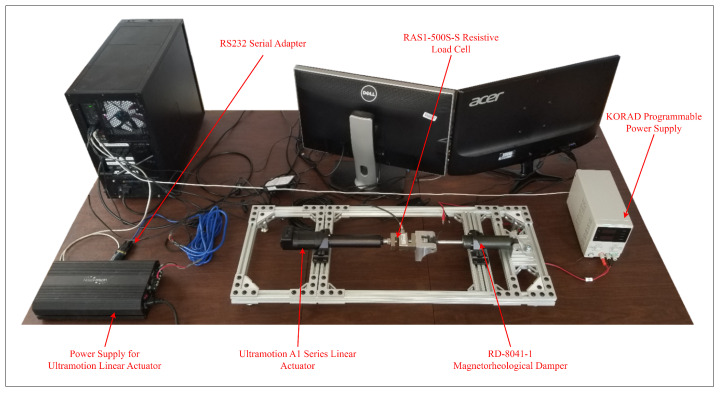
Magnetorheological testing setup used in this study.

The experimental setup was developed at the University of Guelph by the primary author. In order to mathematically model the MR damper, an A1 series linear actuator from Ultra Motion was employed to actuate the damper. An RAS1-500S-S resistive load cell acquired from Loadstar Sensors was used to measure the damping force, and a Korad programmable power supply was used to supply current to the MR damper. Data acquisition and command transmission occurred via RS232 serial communication on a laboratory computer. The components were assembled using an extruded t-slotted aluminum frame, as depicted in Figure 3.

The RD-8041-1 is a linear MR damper with continuous variable damping determined by the yield strength of the MR fluid in response to a magnetic field. The damper responds in less than 15 milliseconds to changes in the magnetic field and can operate at 1 A continuously or 2 A intermittently at 12 Volts DC. The RD-8041-1 is a monotube shock containing high-pressure nitrogen gas (300 psi), which fully extends the piston under no load. At ambient temperatures, the resistance of the coil is 5Ω and at 71 °C, the resistance increases to 7Ω. Extreme temperature changes can drastically alter the performance of the MR damper [34].

The Ultra Motion linear actuator used to drive the MR damper is a standard servo cylinder with an ACME screw to prevent back-drive and operates at a power rating of 180 Watts. The actuator is capable of 445 Newtons of continuous force and 1001 Newtons at its peak with a maximum speed of 178 mm/s. Numerous onboard sensors are employed for the measurement of various states, including position, torque, temperature, and humidity. The position of the linear actuator is measured using the phase index absolute position sensor. This sensor is a multi-turn magnetic encoder with a resolution of 1024 counts per revolution used for absolute position feedback and commutation. The measurement noise covariance of the sensor is discussed in subsequent sections. The torque feedback is calculated using closed-loop current feedback on each motor phase, which may then be subsequently translated into an actuator output force. Utilizing current feedback as a means for output force calculation has been found to be lacking in accuracy, leading to notable discrepancies and noise in the measurements.

In general, a direct relationship exists between motor torque and actuator output force, yet several complicating factors may exert significant influence on this association. Rotational inertial loads, lubricant viscosity, and seal friction can all contribute to output force variability. Factory test data were used in order to convert motor torque into actuator output force. The data are collected on each actuator during the acceptance test procedure (ATP) before leaving the factory [35]. The generated current-force curves exhibit distinctive characteristics for individual actuators. Nevertheless, notable fluctuations in force output persist. To mitigate some of the fluctuations in the torque sensor data, a first-order Butterworth filter was implemented with a cutoff frequency between 0 and 0.05 of the Nyquist rate.

The RAS1-500S-S is a resistive S-Beam load cell capable of measuring both compressive and tensile force measurement. The load cell is made from tool steel and has a capacity of 2224 N and a sample rate of 1000 Hz. The calibration measurement equipment is traceable to NIST via Pacific Calibration Services. This sensor was employed to assess the effectiveness of implementing adaptive filtering strategies on the current feedback of the linear actuator. While the noise covariance of the load cell is 26.535 N, the noise covariance of the Ultra Motion motor torque sensor is 622.407 N. The comparatively high noise distribution of the onboard Ultra Motion motor torque sensor makes it a suitable candidate for applying adaptive filtering strategies.

Force-velocity hysteresis curves have been modeled extensively by [36,37]. However, at low velocities over long stroke lengths, the force of the diaphragm and compressed nitrogen gas is not negligible. Thus, a force-position hysteresis curve was modeled by driving the MR damper at a constant velocity over one full stroke. For the MR model used in this study, the actuator speed was set to 30 mm/s, and the damping force was recorded by the load cell over a stroke length of 57 mm. Approximately 200 strokes (extension and retraction) were used to model the behaviour at each operational mode (normal, over-current, undercurrent). The conditions of the operational modes are discussed below.

There are several different types of faults that can be experienced during MR damper operations. The viscosity of the MR fluid is sensitive to extreme temperatures [33], and the particles in the MR fluid are also subject to degradation over time [38]. However, this study primarily investigates issues arising from faulty power supplies, which alter the current supplied to the MR damper. Undercurrent and over-current fault modes were modeled in addition to the normal operating current. The undercurrent, normal, and over-current operational modes are denoted by a supply current of 0 mA, 120 mA, and 220 mA, respectively.

**Figure 4:**
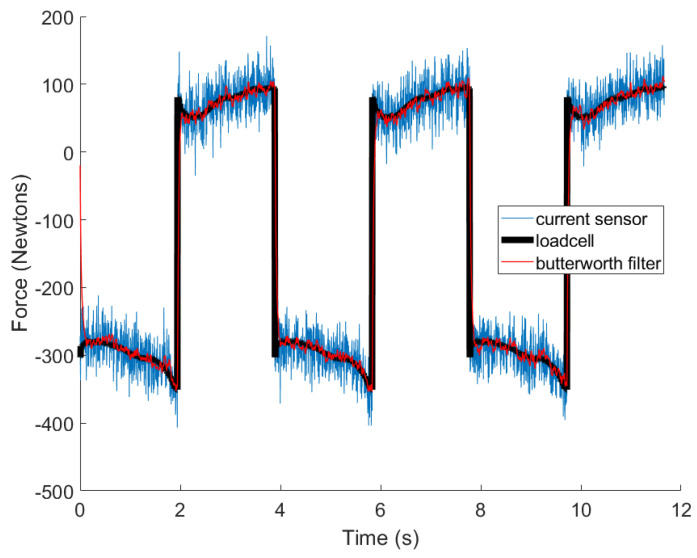
Sample of experimental data used to model the MR damper under normal operating conditions.

A sample of experimental data used to model the MR damper can be seen in Figure 4. The diagram depicts the actuator undergoing constant-speed extension and retraction, with measurements of MR force captured by a load cell and an actuator current sensor. Additionally, the figure demonstrates the application of a first-order Butterworth filter on the actuator current sensor readings to diminish noise before implementing adaptive filtering techniques.

## 5. Magnetorheological Damper Setup

The force-velocity hysteresis of an MR damper has been described in the literature using many different mathematical models such as the nonlinear hysteretic bi-viscous model, polynomial function model, generalized sigmoid hysteresis model, and Bouc-Wen hysteresis model [36]. However, under conditions of low velocities and extended stroke lengths, the force exerted by the diaphragm and the compressed nitrogen gas cannot be disregarded. Consequently, the correlation between the force generated by the MR damper and its position was integrated into established models.

**Figure 5:**
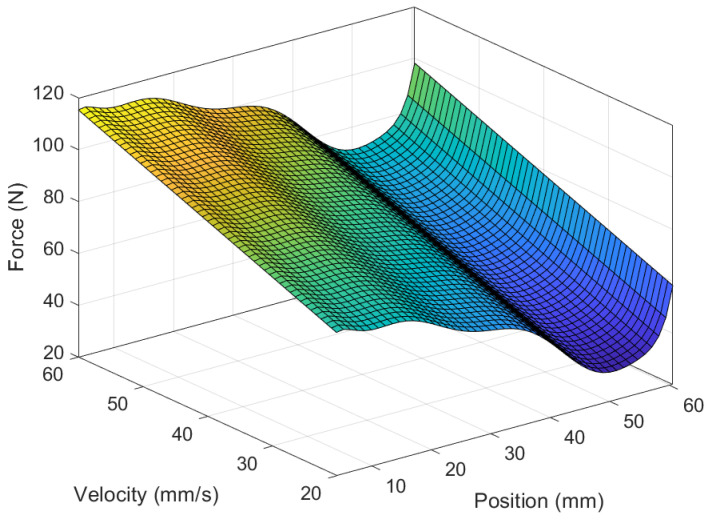
MR force during extension with respect to position and velocity.

**Figure 6:**
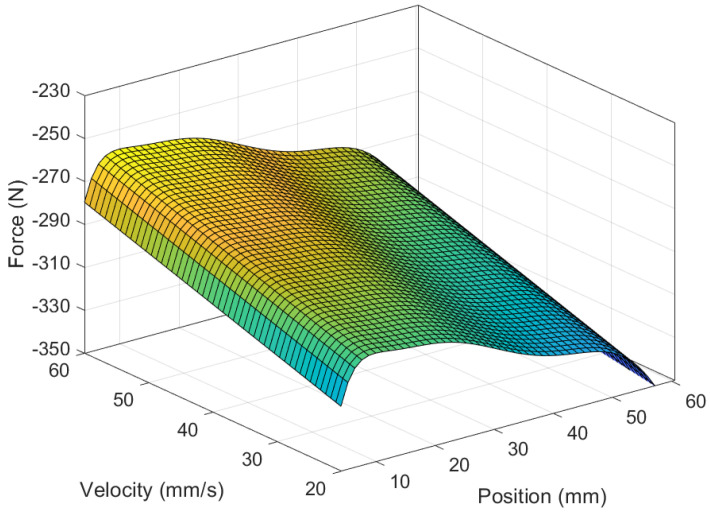
MR force during retraction with respect to position and velocity.

**Figure 7:**
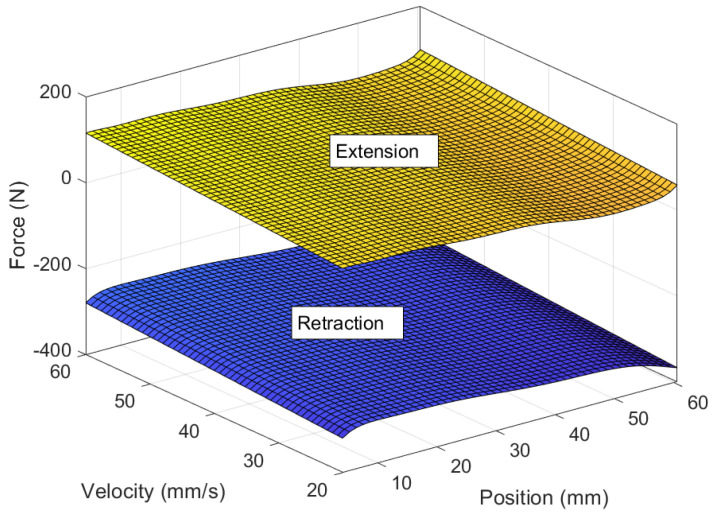
Full MR force model with extension and retraction.

The comprehensive mathematical model of the MR damper computes force based on velocity, position, and the applied current. When maintaining a constant current, the force can be expressed as a function of position and velocity and can be represented as a polynomial surface, as depicted in Figure 5, Figure 6 and Figure 7. Since the experiments were conducted using constant velocity, the model was further reduced to Equation (51).

In this experiment, a ninth-order polynomial model was selected due to its computational efficiency when implementing model-based filters such as EKF, UKF, and ESIF without compromising model accuracy. The basic polynomial hysteresis function is presented as follows:

(50) $$f_{h}=\sum_{k=0}^{n}a_{k}y^{k};\;n=9,$$

where *y* is the position of the MR piston, $a_{k}$ is the polynomial coefficient constant, which is experimentally obtained, *k* represents the polynomial exponent, and *n* represents the polynomial order [36]. The velocity (direction) of the piston determines whether the damping force follows the upper or lower hysteresis curve, as shown as follows [36]:

(51) $$f_{d}=\left\{\begin{array}{cc} \sum_{k=0}^{9}a_{uk}y^{k} & ;\;\dot{y}<0\\ \sum_{k=0}^{9}a_{dk}y^{k} & ;\;\dot{y}>0\\ \sum_{k=0}^{9}\frac{1}{2}\left(a_{uk}+a_{dk}\right)y^{k} & ;\;\dot{y}=0 \end{array}\right.,$$

where $a_{uk}$ and $a_{dk}$ are the lower and upper polynomial coefficients, respectively. Convergence of the two polynomial functions near the extremities is ensured by averaging the lower and upper polynomial functions when the piston velocity changes direction or is equal to 0 mm/s [36]. The coefficients of the polynomial model are given in Table 1.

**Figure 8:**
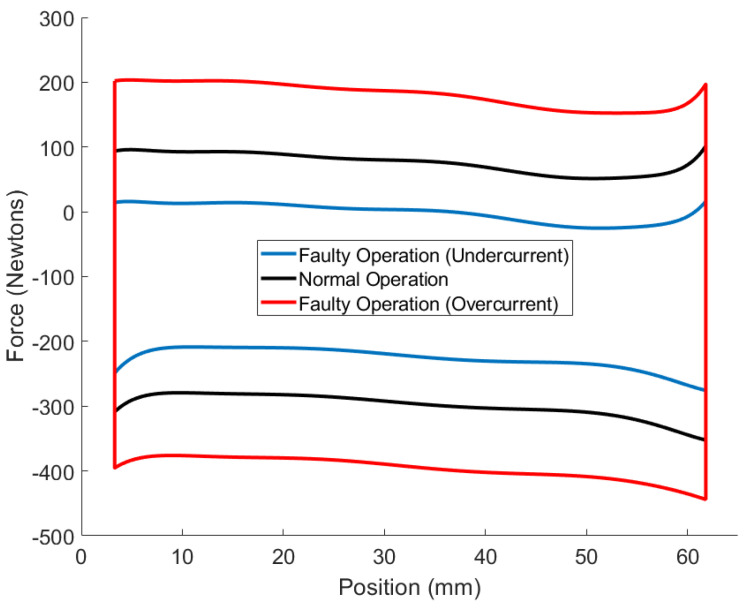
MR damping force with respect to position when piston velocity is set to 30 mm/s.

The models shown in Figure 8 depict the force–position hysteresis relationship of the MR damper at a velocity of 41.5 mm/s. This represents a cross-section of Figure 6 at the specified velocity. The data points were fitted using Equation (51) to obtain the polynomial coefficients in Table 1. The norm of the residuals for each data set to their polynomial models are [12.086, 8.1279], [6.794, 8.070], and [7.367, 13.693] for the undercurrent, normal, and overcurrent modes, respectively. The first number represents the upper polynomial curve, while the second represents the lower polynomial curve.

The discretized state space equations can be written as follows:

(52) $$x_{1,k+1}=x_{1,k}+T\cdot x_{2,k},$$

(53) $$x_{2,k+1}=x_{2,k},$$

(54) $$x_{3,k+1}=\left\{\begin{array}{cc} \sum_{k=0}^{9}a_{uk}x_{1,k} & ;\;x_{2,k}<0\\ \sum_{k=0}^{9}a_{dk}x_{1,k} & ;\;x_{2,k}>0\\ \sum_{k=0}^{9}\frac{1}{2}\left(a_{uk}+a_{dk}\right)x_{1,k} & ;\;x_{2,k}=0 \end{array}\right.,$$

where $x_{1}$, $x_{2}$, $x_{3}$, are the position, velocity, and force of the MR damper and *T* is the sampling rate.

The system and measurement noise covariance matrices are defined respectively, as follows, based on factory testing:

(55) $$Q=R\times 10^{-1},$$

(56) $$R=\left[\begin{array}{ccc} 5.5134\times 10^{-4} & 0 & 0\\ 0 & 7.797\times 10^{-4} & 0\\ 0 & 0 & 622.407 \end{array}\right].$$

The system noise was not measured directly but was assumed to be one magnitude smaller than the measurement noise.

## 6. Results and Discussion

**Figure 9:**
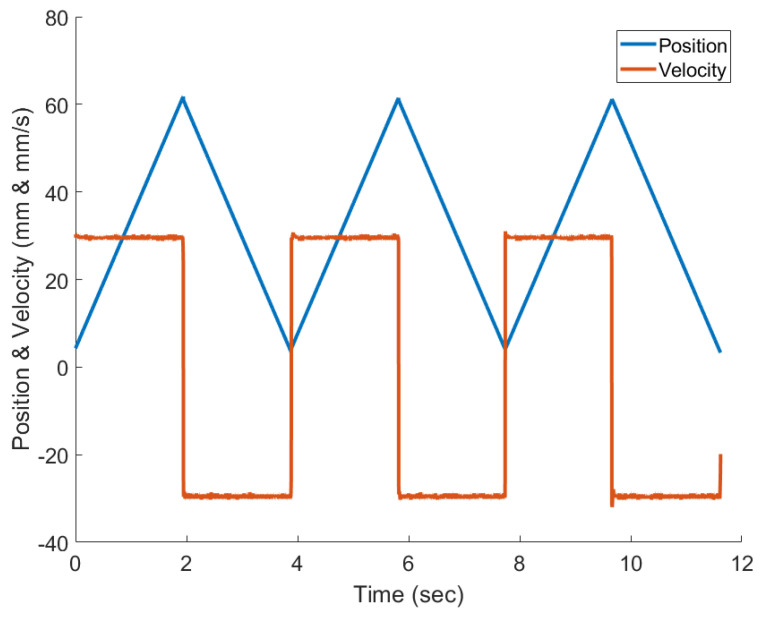
Sample of experimental data used to model the MR damper under normal operating conditions.

The linear actuator drove the MR damper for a total of 11.62 s with constant velocity (30 mm/s) during extension and retraction. The position and velocity profile captured by the actuator encoder can be seen in Figure 9. The initial current of 120 mA was applied to the MR damper, which represents normal operation. The MR damper was allowed to fully extend and retract before an overcurrent fault (220 mA) was introduced at 3.86 s. After another full period of motion, an undercurrent fault (0 mA) was introduced to the MR damper at 7.73 s before completing a final extension and retraction.

The fixed boundary layer applied in the ESIF was tuned based on minimizing the force state estimation error. The smoothing boundary layer widths are given by the following:

(57) $$\delta =\left[\begin{array}{ccc} 5.5134\times 10^{-4} & 0 & 0\\ 0 & 7.797\times 10^{-4} & 0\\ 0 & 0 & 80 \end{array}\right].$$

For all estimation strategies, the initial conditions were set to the following:

(58) $${\hat{x}}_{0}={\left[4.2788\;30.2793\;-303.0187\right]}^{T},$$

(59) $$P_{0|0}=10*Q.$$

For the experiments conducted in this paper, it is assumed that the MR damper operates normally 65% of the time and has an equal likelihood of experiencing an undercurrent or overcurrent fault. The initial mode probability $\mu_{i,0}$ is given as follows:

(60) $$\mu_{i,0}={\left[0.65\;0.175\;0.175\right]}^{T}.$$

Based on experimental procedures, the mode transition matrix $p_{i,j}$ is defined by a 3 by 3 diagonal matrix with 0.65 on the diagonal and 0.175 on the off-diagonal. This transition matrix signifies that there is a 65% probability that the system will remain in the current mode. For example, if the system is experiencing normal operation, there is a 65% chance the system will continue to undergo normal operation in the next time step.

**Figure 10:**
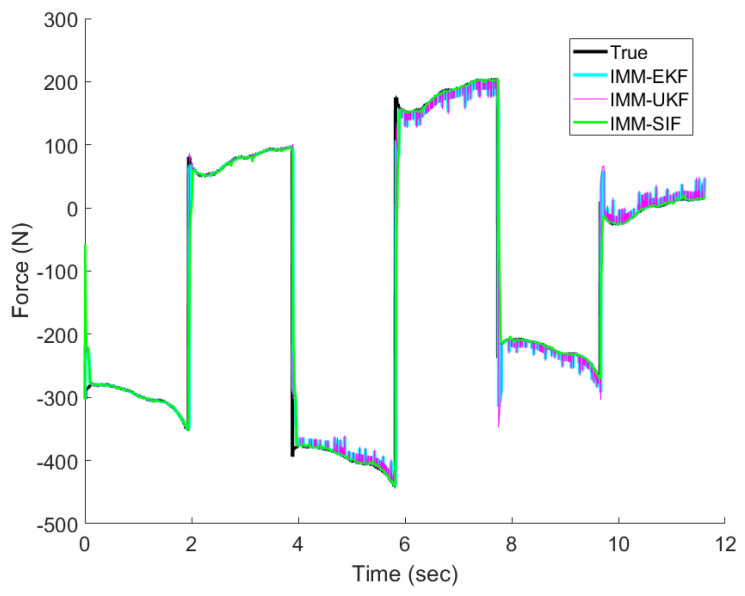
Force estimation of the MR damper undergoing mixed operation with normal, overcurrent, and undercurrent modes.

As described previously, the experiment consisted of a test in which all three modes (normal, overcurrent, undercurrent) were experienced. Following one actuation period in a specific mode, the system transitioned to the next mode in sequence until all three modes were introduced. Figure 10 shows the results of the IMM-EKF, IMM-UKF, and IMM-ESIF for estimating the force exerted by the MR damper during testing.

The root mean squared error (RMSE) for each estimator was calculated as follows:

(61) $$RSME=\sqrt{\frac{\sum_{n=1}^{n}\left(x_{i}-{\hat{x}}_{i}\right)}{n}},$$

where *n* is the number of steps. The values shown in Table 2 and Table 3 are the average RMSE of the 20 separate trials similar to the one shown in Figure 10. The order in which the modes were experienced was randomized for each trial.

The IMM-EKF, IMM-UKF, and IMM-ESIF perform comparatively well when the MR is in normal operation. As shown in Table 2, the IMM-ESIF performs slightly better than the IMM-EKF and IMM-UKF under normal operation. However, the benefit of the increased robustness is demonstrated in Table 3, which shows the RMSE for mixed operation. In the presence of faults and modeling uncertainty, the IMM-ESIF shows a clear advantage over its counterparts. There is an 83.7% improvement over the IMM-EKF and an 89.4% improvement over the IMM-UKF. It is interesting to note that while the UKF generally performs better than the EKF for highly nonlinear systems, the EKF outperformed the UKF during mixed operations.

**Figure 11:**
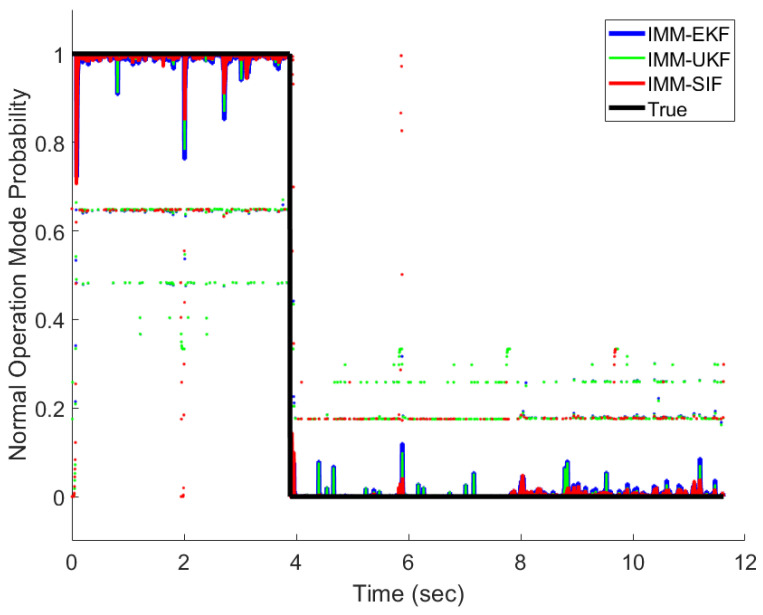
Normal operation mode probability.

**Figure 12:**
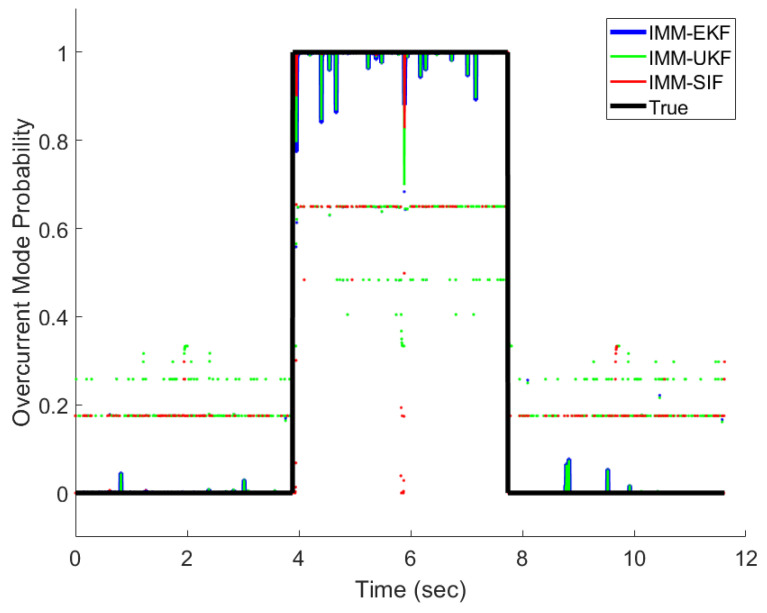
Overcurrent fault mode probability.

**Figure 13:**
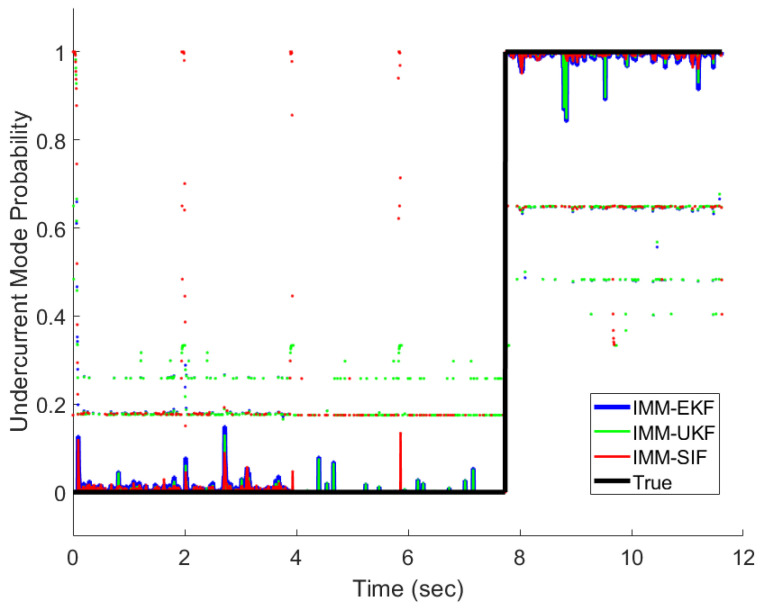
Undercurrent fault mode probability.

The IMM-EKF, IMM-UKF, and IMM-ESIF were all able to properly detect the mode probabilities with varying degrees of confidence. Figure 11, Figure 12 and Figure 13 show the mode probabilities calculated by each estimation strategy. In order to clearly depict the mode probabilities, the overall trends are shown as solid lines, while spikes in the mode probability are represented as dots. The mode probabilities show that the IMM-ESIF misclassifies the correct mode when the velocity of the MR damper changes direction. However, the overall classification accuracy of the IMM-ESIF is higher than its counterparts.

A value “1” for a mode probability refers to a 100% confidence that the system is experiencing that mode, while a “0” refers to a probability of 0%. Table 4, Table 5 and Table 6 illustrate the confusion matrices for each estimator that are commonly used in fault detection and diagnosis. The vertical axis typically represents the predicted mode, while the horizontal axis represents the actual mode being experienced by the MR damper.

The presented confusion matrices illustrate that the IMM-EKF, IMM-UKF, and IMM-ESIF models successfully predicted the correct operational mode with a notable degree of confidence. Specifically, it is observed that the classification accuracy for normal operation was relatively lower in comparison to the other modes. This discrepancy can be attributed to the fact that the damping force associated with normal operation falls within the range between the overcurrent and undercurrent modes, as depicted in Figure 8. Likewise, the classification of overcurrent fault had the highest accuracy because it has greater separation from the normal operation than the undercurrent fault. The IMM-UKF had slightly higher classification accuracy than the IMM-EKF. However, the IMM-ESIF shows a 4–5% higher accuracy when classifying the correct mode when compared to the IMM-EKF and IMM-UKF. Overall, the IMM-ESIF showed significant improvement in both estimation accuracy (RMSE) and classification (confusion matrix) when compared to the IMM-EKF and IMM-UKF.

## 7. Conclusions

This paper introduced a novel model-based estimator that combined the IMM strategy with the relatively new ESIF. The novel estimator, referred to as the IMM-ESIF, was applied to an MR damper for force estimation and fault detection. The experiments involved three distinct operational modes: normal operation, an overcurrent fault, and an undercurrent fault. It is noteworthy that the damping behaviour of the MR damper is significantly influenced by the supplied current, making prompt identification of power supply faults crucial. During normal operation, the IMM-ESIF demonstrated performance on par with other well-established Kalman-based strategies. However, when the MR damper operated under mixed conditions (both normal and faulty operation), the IMM-ESIF outperformed both IMM-EKF and IMM-UKF. In fact, the IMM-ESIF exhibited a substantial reduction in estimation error, ranging from 80% to 90% compared to its counterparts. Additionally, it displayed a 4% to 5% improvement in correctly classifying operational modes, resulting in fewer misclassifications compared to other estimators. The IMM-ESIF emerges as a promising alternative to existing IMM estimation strategies. In light of the outcomes achieved by the proposed IMM-ESIF model-based estimator, the trajectory for future research endeavors presents a rich landscape for exploration and refinement. Firstly, an avenue for investigation lies in the comprehensive examination of additional fault scenarios within MR dampers, such as those related to MR fluid degradation, to ascertain the robustness and versatility of the IMM-ESIF across a spectrum of potential challenges. This research could delve into the development of tailored fault detection strategies, leveraging the inherent strengths of the IMM-ESIF in capturing nuanced variations in damper behaviour. Furthermore, the exploration of alternative nonlinear formulations of the SIF, coupled with a thorough integration into the IMM framework, holds the promise of further enhancing estimation accuracy. The synergistic fusion of advanced signal processing techniques and machine learning methodologies may be explored to push the boundaries of estimator performance, especially in scenarios involving complex and dynamic interactions. Additionally, comparative studies involving a broader array of well-established estimation strategies can be undertaken to establish a more nuanced understanding of the IMM-ESIF’s relative advantages and limitations. By addressing these facets, future research endeavors can contribute significantly to the evolution of model-based estimators.

## Figures and Tables

**Table 1:** Experimentally obtained coefficients for the polynomial for the magnetorheological damper model.

Polynomial Coefficient	Undercurrent (0 mA)	Normal Operation	Overcurrent (220 mA)
$a_{u0}$	−362.5338	−402.2871	−455.7308
$a_{u1}$	55.6247	46.6965	29.2484
$a_{u2}$	−8.3330	−7.2734	−4.2088
$a_{u3}$	0.6791	0.6116	0.3078
$a_{u4}$	−0.0333	−0.0309	−0.0125
$a_{u5}$	0.0010	9.7782 $\times \;10^{4}$	2.8271 $\times \;10^{4}$
$a_{u6}$	−2.0429 $\times \;10^{5}$	−1.9756 $\times \;10^{5}$	−3.2121 $\times \;10^{6}$
$a_{u7}$	2.5395 $\times \;10^{7}$	2.4911 $\times \;10^{7}$	9.3169 $\times \;10^{9}$
$a_{u8}$	−1.8183 $\times \;10^{9}$	−1.7990 $\times \;10^{9}$	1.3375 $\times \;10^{10}$
$a_{u9}$	5.7430 $\times \;10^{12}$	5.7019 $\times \;10^{12}$	−9.3501 $\times \;10^{13}$
$a_{d0}$	−27.3674	47.9106	167.3362
$a_{d1}$	28.4778	30.7247	24.0020
$a_{d2}$	−7.1661	−7.5816	−6.1759
$a_{d3}$	0.8976	0.9404	0.8009
$a_{d4}$	−0.0633	−0.0663	−0.0587
$a_{d5}$	0.0027	0.0028	0.00264
$a_{d6}$	−6.8046 $\times \;10^{5}$	−7.1970 $\times \;10^{5}$	−6.7311 $\times \;10^{5}$
$a_{d7}$	1.0341 $\times \;10^{6}$	1.1028 $\times \;10^{6}$	1.0519 $\times \;10^{6}$
$a_{d8}$	−8.5880 $\times \;10^{9}$	−9.2442 $\times \;10^{9}$	−8.9620 $\times \;10^{9}$
$a_{d9}$	3.0007 $\times \;10^{11}$	3.2615 $\times \;10^{11}$	3.2055 $\times \;10^{11}$

**Table 2:** Tabulated RMSE of various estimation strategies under normal operation.

Estimation Strategy	RMSE (Newtons)
IMM-EKF	2.37
IMM-UKF	2.36
IMM-ESIF	1.97

**Table 3:** Tabulated RMSE of various estimation strategies under mixed operation.

Estimation Strategy	RMSE (Newtons)
IMM-EKF	17.52
IMM-UKF	19.20
IMM-ESIF	2.04

**Table 4:** IMM-EKF confusion matrix.

	Actual Normal Operation	Actual Overcurrent Fault	Actual Undercurrent Fault
**Predicted Normal Operation**	88.75%	4.86%	5.62%
**Predicted Overcurrent Fault**	4.49%	90.58%	5.08%
**Predicted Undercurrent Fault**	6.76%	4.55%	89.30%

**Table 5:** IMM-UKF confusion matrix.

	Actual Normal Operation	Actual Overcurrent Fault	Actual Undercurrent Fault
**Predicted Normal Operation**	88.99%	4.84%	5.43%
**Predicted Overcurrent Fault**	4.48%	90.61%	5.07%
**Predicted Undercurrent Fault**	6.53%	4.55%	89.50%

**Table 6:** IMM-ESIF confusion matrix.

	Actual Normal Operation	Actual Overcurrent Fault	Actual Undercurrent Fault
**Predicted Normal Operation**	93.78%	2.66%	2.09%
**Predicted Overcurrent Fault**	1.26%	94.58%	1.55%
**Predicted Undercurrent Fault**	4.97%	2.76%	96.36%

## Data Availability

The data presented in this study are available on request from the corresponding author.
